# The mediating role of the connection with nature in the relationship between behavioral problems and emotion regulation in early childhood

**DOI:** 10.3389/fpsyg.2025.1583794

**Published:** 2025-10-30

**Authors:** Melike Aykut, H. Gözde Ertürk Kara

**Affiliations:** Kocaeli University, İzmit, Türkiye

**Keywords:** early childhood, connection to nature, emotion regulation, behavior problems, mediation analysis

## Abstract

**Introduction:**

This study aims to investigate the mediating role of connection to nature in the relationship between behavioral problems and emotion regulation in preschool children.

**Methods:**

A cross-sectional research design was used to collect data from a single time point. The study included 299 children (M < sub > age</sub > = 60.84 months, SD = 3.07). Children’s connection to nature was measured using the Disposition Toward Connecting with Nature Scale, emotion regulation skills were assessed with the Emotion Regulation Scale, and behavioral problems were evaluated using the Behavior Rating Scale.

**Results:**

The results indicated that there was a strong, statistically significant negative correlation between children’s connection to nature and behavioral problems (*r* = −0.884, *p* < 0.01), and a positive correlation with emotion regulation skills (*r* = 0.831, *p* < 0.01). Additionally, emotion regulation skills and behavioral problems demonstrated a strong, significant negative correlation (*r* = −0.772, *p* < 0.01). Bootstrap mediation analysis (PROCESS Macro Model 4) revealed that connection to nature partially mediated the relationship between behavioral problems and emotion regulation skills [indirect effect = −0.0231, BootSE = 0.0029, 95% CI (−0.0287, −0.0174)]. The direct effect of behavioral problems on emotion regulation remained significant (c’ = −0.028, *p* < 0.01), confirming partial mediation. This finding indicates that connection to nature is not the sole determining factor for children’s emotion regulation, but it plays a supportive role in enhancing their emotion regulation skills during this process. It has been observed that connection to nature can serve as a supportive factor for emotion regulation and may help children with behavioral issues cope more effectively with challenges in emotional management.

**Discussion:**

These findings underscore the potential benefits of integrating nature-based activities into strategies that support children’s emotional development and may assist those with behavioral challenges.

## Introduction

Emotion regulation refers to an individual’s ability to identify, evaluate, and adjust their emotions in a flexible manner that is contextually appropriate and socially acceptable (Braund and Timmons, 2021; Malhi et al., 2017; Thompson, 1994). Developing these skills in early childhood is critically important, as children benefit from support in learning strategies to manage their emotions effectively (Cole et al., 2009; Thümmler et al., 2022). Emotion regulation in children is crucial for long-term psychological well-being, social adjustment, and moral development (Blair et al., 2016; Di Maggio et al., 2016; Indarwati et al., 2019). These foundational insights suggest that supporting emotion regulation from early childhood is crucial not only for individual well-being but also for social and moral development, highlighting the importance of identifying factors that may enhance or hinder this process.

Classical developmental theories provide insight into the emergence of emotion regulation. Piaget (1965) emphasized gradual development of social understanding through peer interactions, while Kohlberg (1969) described the progression from authority-based rules to complex social contracts. Hoffman’s (2000) empathy theory highlights that sensitivity to others’ emotional states forms the foundation for empathic guilt, prosocial behaviors, and emotion regulation. Empirical evidence supports these perspectives: even infants demonstrate early social-cognitive skills such as gaze-following, emotion recognition, and nascent moral evaluations (Geraci et al., 2024; Geraci et al., 2023; Kanakogi et al., 2013). This indicates that empathy and moral sensitivity, including environmental awareness, develop from infancy and may influence the emergence of emotion regulation skills.

Behavioral problems can disrupt social adaptation and emotion regulation development (Najmussaqib and Mushtaq, 2023; Yoleri, 2013). Emerging evidence suggests that connection to nature may serve as a protective factor, supporting attention, stress regulation, and social–emotional skills in children (Lanza et al., 2022; Pritchard et al., 2020). Children with stronger emotional attachment to nature demonstrate enhanced self-awareness, self-management, and relationship skills (Ives et al., 2017), indicating that nature connectedness could mediate the impact of behavioral problems on emotion regulation.

## Literature review

### Behavioral problems and emotion regulation across development

Research shows that ER skills are influenced by both biological and environmental factors from early life. In infancy and toddlerhood, ER development is closely linked to caregiver-infant interactions. Bowlby’s (1969) attachment theory and Ainsworth et al.’s (1978) work on secure and insecure attachment emphasize that sensitive and responsive caregiving facilitates adaptive emotion regulation, whereas inconsistent or unsupportive caregiving can contribute to early behavioral difficulties. Supporting this, İşcanoğlu et al. (2024) found that unsupportive maternal responses exacerbate behavioral difficulties, especially in children with low ER capacity.

Neurobiological evidence also highlights early differences in ER-related brain activity. Etkin et al. (2011) reported that difficulties in emotion regulation are associated with low activity in dorsal cortical regions, particularly the anterior cingulate cortex (ACC). Mehmood et al. (2021) corroborate these findings, noting that neurocognitive differences are observable before school age. Granic and Lamey (2002) further demonstrate that inflexible parent–child interactions in aggressive children hinder ER development, emphasizing the interplay between relational experiences and behavioral outcomes.

There is ongoing debate about the directionality of the relationship between emotion regulation and behavioral problems. Some studies suggest that poor ER is a precursor to behavioral difficulties (Crespo et al., 2017; Eisenberg et al., 2001), whereas others show that behavioral problems can disrupt the development of adaptive ER skills, creating a negative feedback loop (Mehmood et al., 2021; Wells et al., 2020). Despite these established links, research on the mechanisms that mediate or mitigate this relationship remains limited. This study aims to address this gap by examining one such pathway: how behavioral problems may impair ER, and how connection to nature may buffer this effect.

### Behavioral problems and connection to nature

Connection to nature has been shown to support emotional and behavioral regulation in children. Louv (2008) argues that time spent in nature enhances emotional regulation and alleviates behavioral problems through calming effects. Similarly, Kuo and Taylor (2004) found that children who spend time in green spaces exhibit fewer attention deficits, regardless of hyperactivity levels. Even infants and toddlers show preferences for natural stimuli and respond positively to nature-supportive environments, suggesting early exposure may influence developmental trajectories (Geraci et al., 2023).

Recent environmental changes, including reduced time in nature and increased screen exposure, may hinder these beneficial interactions (Heerwagen and Orians, 2002; Larson et al., 2011; Radesky et al., 2015). Additionally, children with behavioral difficulties may be less inclined to engage with nature effectively. Poor impulse control or introverted behavior can limit children’s ability to focus or interact with natural environments (Driessnack, 2009; Hechter and Fife, 2019). Understanding these dynamics is crucial for designing interventions that support emotional development.

### Connecting with nature and emotion regulation

Early interventions are essential for teaching adaptive strategies for emotion recognition and regulation (Wakschlag et al., 2019). Children with strong ER skills use positive strategies such as cognitive restructuring, substitution, and self-soothing (Gross, 2002). Nature-based interventions reinforce these strategies from infancy through preschool age. For example, a systematic review by Johnstone et al. (2022) found that nature-based early childhood education positively influenced self-regulation, social skills, social–emotional development, nature connectedness, and play. Ernst and Stelley (2024) report similar benefits for hot executive function in preschoolers, and Bastianello et al. (2024) demonstrate that short-term outdoor interventions improve stress regulation and socioemotional skills.

Overall, interacting with natural environments promotes calmness, attentional control, and emotional balance, providing a developmentally supportive context for emotion regulation skill acquisition (Kaplan and Kaplan, 1989; Kuo, 2015; Ríos-Rodríguez et al., 2024). Integrating insights from developmental psychology, early childhood research, and nature-based interventions allows for a comprehensive understanding of how behavioral problems, emotion regulation, and nature connectedness interact during preschool years.

Research Aim and Hypotheses:

This study investigates whether connection to nature mediates the relationship between behavioral problems and emotion regulation in preschool children. The main research question is: How do behavioral problems shape children’s emotion regulation skills by affecting their level of connection to nature? The hypotheses are:

*H1*: Behavioral problems negatively affect children’s connection to nature.

*H2*: Connection to nature positively influences emotion regulation skills.

*H3*: The effect of behavioral problems on emotion regulation occurs indirectly through connection to nature.

## Method

### Research model

This study is designed as a cross-sectional correlational study, aiming to reveal the relationship between variables (Fraenkel et al., 2011). In this context, attempts are being made to reveal the relationships between children’s connection to nature, behavior problems, and emotion regulation skills. The dependent variable of this research is emotion regulation, while behavior problems are the independent variable, and connection to nature is the mediator variable.

### Study group

The study group was selected via convenience sampling, while also considering the variability among children based on socioeconomic status. Therefore, preschools that can be accessed by the researcher were visited in İzmit (middle to upper socioeconomic status), and Derince (low socioeconomic status) districts. Participants were selected from those whose teachers and managers approved their participation in the study. The mean age is 60.84 months (SD = 3.07). A total of 299 children were reached. Based on their school types, 59.8% of the students of the study group are from standalone preschools, which operate independently, while 40.2% of the study group is from preschool classrooms integrated within primary schools.

### Measures

#### Disposition toward Connecting with Nature Scale

Developed by Engin and Demiriz (2022), this scale was completed by the children’s teachers to measure 60-to-72-month-old children’s connection to nature. While developing the scale, validity studies were conducted based on expert opinions and factor analytic techniques. Exploratory factor analysis revealed a single- factor structure with 23 items. Factor loadings ranged between 0.38 and 0.66. Confirmatory factor analysis results show that the structure is confirmed (χ2/df = 1.367; CFI > = 0.90; TLI > = 0.90; RMSEA <= 0.08). For reliability purposes, McDonald Omega (*ω* = 0.989) and test–retest reliability coefficients (0.852) were studied. Both statistics indicated reliable scale scores. The 3-point Likert scale is used for answers (1, never, 2, some, 3, much).

#### Behavior Rating Scale

Originally developed by Behar and Stringfield (1974), this scale was completed by the children’s teachers to detect the emotional challenges of preschool students. A Total of 30 items were used in the scale measuring three main subscales, labeled as Hostile-Aggressive, Anxious-Fearful, and Hyperactive-Distractible. Validity and reliability studies were conducted by criterion validation, by interrater reliability, and by test–retest reliability and reported to prove that the scale produces valid and reliable scores. Kanlıkılıçer (2005) studied the scale for adaptation purposes. The scale produced identical subscales in the Turkey sample. However, some items are excluded from the scoring due to low factor loading (i30). Item total score correlations range between 0.184 and 0.718. Exploratory factor analysis revealed 47% of the children’s behavior problems. Subscale correlations are between 0.412 and 0.693. The Cronbach Alpha reliability coefficient is 0.920 for the adaptation studies. The rating is 0 for not correct, 1 for sometimes correct, and 2 for absolutely correct.

#### Emotion Regulation Scale

This scale was developed by Shields and Cicchetti (1997) with 24 items under two subscales. The structure of the scale, which was adapted by Yagmurlu and Altan (2010), was also validated in the Turkish sample. The scale was completed by the children’s teachers. For this study, the emotion regulation subscale is used. This subscale consists of 8 items. The total score for this subscale is calculated by summing the responses to its 8 items, with higher scores indicating better emotion regulation skills. The internal consistency reliability obtained in the adaptation study were calculated as 0.75 for the mother’s rating and 0.84 for the teacher’s rating. The rating for items is as follows: 1: never/seldom, 2: sometimes, 3: often, 4: almost all the time. Item 12 is not included in the subscale total.

#### Validity and the reliability of the measurement tools for study group

The validity evidence of the Disposition Toward Connecting with Nature Scale for the study group was established through confirmatory factor analysis. Accordingly, the structure of the scale produces valid results in the study group (CFI > = 0.995; TLI = 0.995; SRMR = 0.039). For reliability, in addition to the McDonald Omega coefficient, reported in the development process of the scale, Cronbach’s Alpha internal consistency coefficient, which is frequently preferred in literature, was also calculated. Both coefficients indicated a high level of reliability (*ω* = 0.983; *α* = 0.981).

The study of the validity of the Behavior Rating Scale for this study group is conducted using confirmatory factor analysis. The data fit indices proved that the scale has its validity in the Turkish sample as well (CFI = 0.994; TLI = 0.994; SRMR = 0.054). Standardized factor loadings range from 0.538 to 0.979. Cronbach’s Alpha internal consistency coefficient is calculated as 0.978. These psychometric statistics proved that the scale has valid and reliable outcomes for the sample of this study.

The confirmatory factor analysis results of the Emotion Regulation subscale showed a good fit in the sample of this study (CFI = 0.992; TLI = 0.988; SRMR = 0.078). The standardized factor loadings range from 0.201 to 0.986. The Cronbach’s Alpha internal consistency reliability coefficient is found to be 0.84. Therefore, it is concluded that the scores are valid and reliable for the study group.

### Data collection

Before data collection, approval was obtained from the committee of the authors’ university. Following receipt of the ethical approval letter, data was collected between April 2024 and November 2024 by teachers. First, teachers were informed about the purpose of the study and data collection tools. Afterwards, detailed explanations were given for each data collection tool. Teachers were informed about the key issues in order to standardize the data collection process. It was emphasized that the teacher should fill in each scale for each student with great care and attention in order to obtain valid and reliable results.

### Data analysis

**Figure 1:**
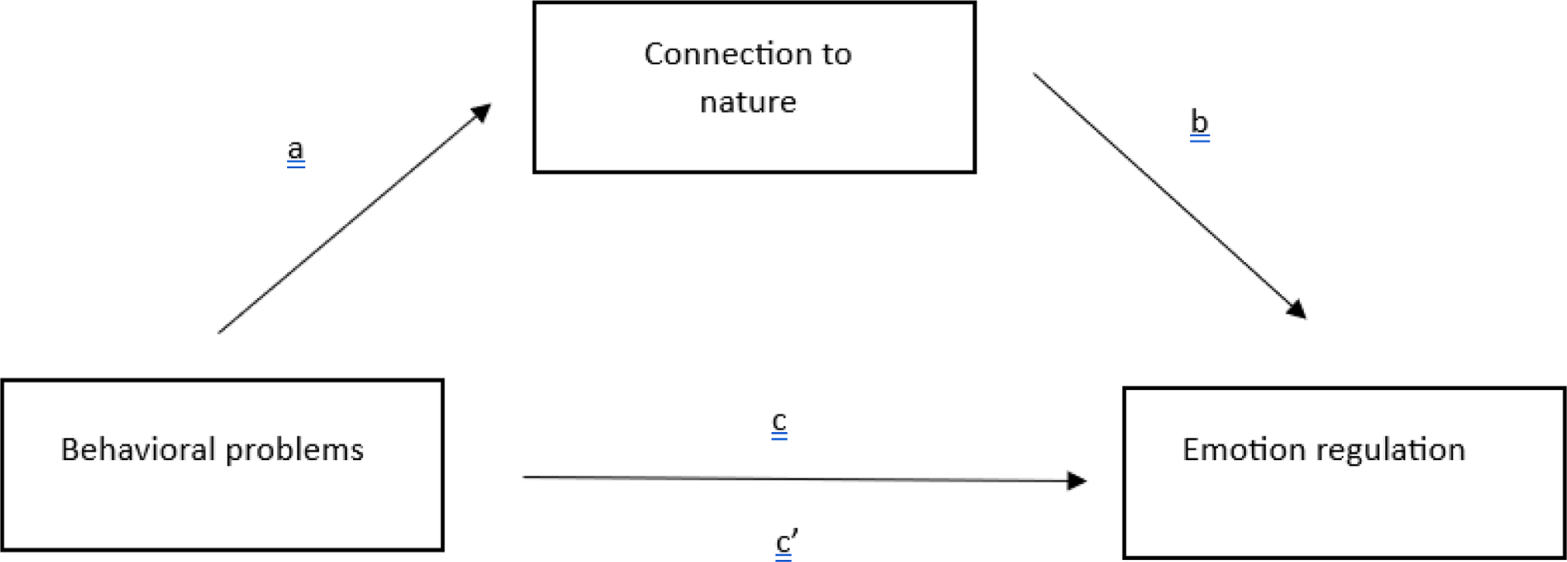
Proposed mediation model.

Before analysis, the data set was examined for missing data. No missing values were observed in the data set. Before proceeding to the mediation analysis, conducted to test the hypothesis of the study, the assumptions of the technique were examined. To test mediation, the causal step approach suggested by Hayes (2022) is followed. Considering the criticisms of the related technique in recent years, bootstrap results of the indirect effects are also reported. The proposed model is presented in Figure 1.

### Findings

Before answering the research hypothesis, the distributions of the scores obtained from the data collection tools for the study group were examined. The relevant distributions are given in Table 1.

**Table 1:** Descriptive statistics for data collection tools.

Scales	Average	Sd	Skewness	Kurtosis
Disposition Toward Connecting with Nature Scale	58.818	12.564	−1.031	−0.088
Behavior Rating Scale	11.377	13.697	1.092	0.137
Emotion Regulation Scale	2.889	0.525	−0.286	−1.189

When examined, two observations from the Behavior Rating Scale indicated extreme values of the standardized z scores. Therefore, those two values are excluded from the data set. All total scores are in line with the limits of normal distribution for skewness and kurtosis values. Table 2 shows the correlations between Disposition Toward Connecting with Nature Scale, Behavior Rating Scale, and Emotion Regulation Scale scores.

**Table 2:** Correlations between scale scores.

Scales	Disposition toward connecting with nature scale	Behavior rating scale	Emotion regulation scale
Disposition Toward Connecting with Nature Scale	1	−0.884**	0.831
Behavior Rating Scale	−0.884**	1	−0.772**
Emotion Regulation Subscale	0.831**	−0.772**	1

**<0.01.

Table 2 reveals the relationships between scale scores. Accordingly, the Disposition Toward Connecting with Nature Scale is negatively correlated with the Behavior Rating Scale (*r* = −0.884; *p* < 0.01). This correlation is strong and statistically significant. On the other hand, a strong positive and statistically significant relationship is found between Disposition Toward Connecting with Nature Scale and Emotion Regulation scores. The last correlation revealed is between emotion regulation and the Behavior Rating Scale. It is found to be a strong negative and statistically significant relationship.

### Findings for the hypothesis

**Figure 2:**
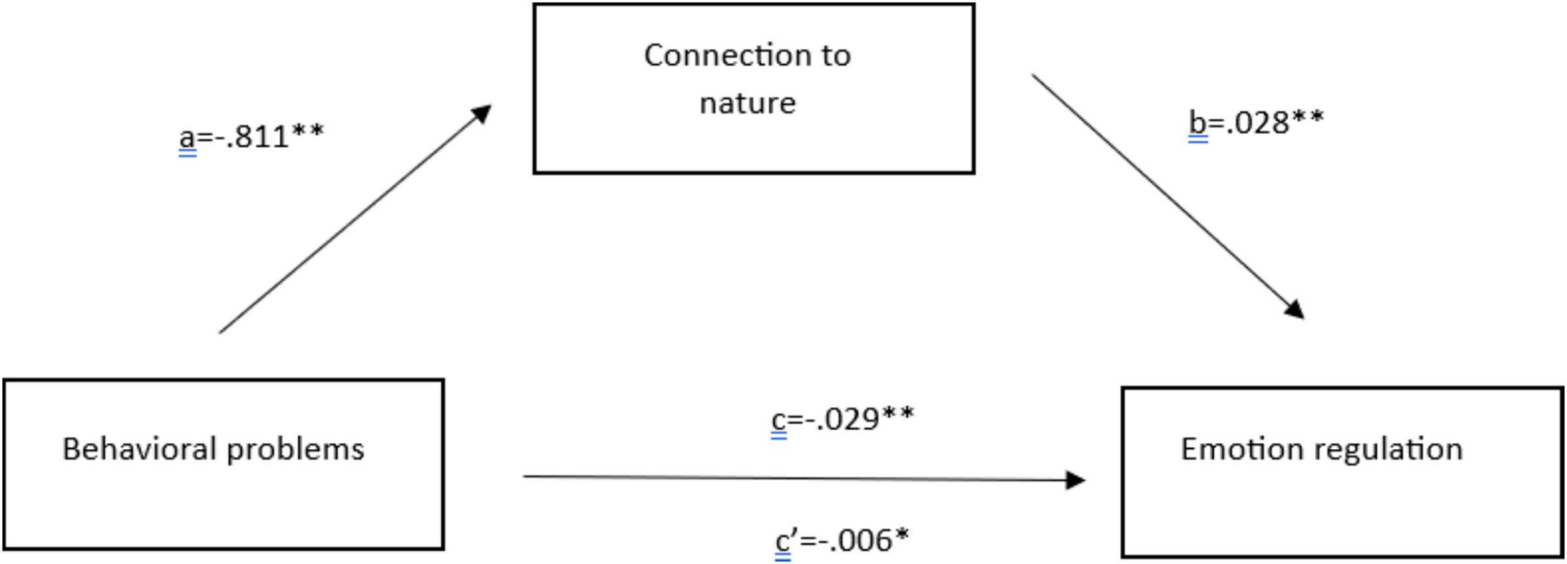
Mediation models with path coefficients.

To test the mediation role of connection to nature in the relationship between behavioral problems and emotion regulation, the HAYES PROCESS Macro Model 4 was used. Additionally, bootstrap results were reported. Figure 2 shows the statistics based on the causal step approach.

*H1*: Behavioral problems negatively affect children’s level of connection to nature (coefficient a).

Coefficient a is calculated as −0.811, indicating that a one-unit change in the behavioral problems score will result in a decline of 0.811 points in the nature scores. This relationship is found to be statistically significant (*p* < 0.01). Therefore, the first hypothesis is accepted.

*H2*: Connection to nature positively influences emotion regulation skills.

The path between connection to nature and emotion regulation is coefficient b in Figure 2. Accordingly, b is 0.028, meaning that one unit change in connection to nature scores will result in a 0.028 points increase in emotion regulation scores. The second hypothesis is accepted.

*H3*: The effect of behavioral problems on emotion regulation occurs indirectly through connection to nature.

To test the final hypothesis, both c and c’ coefficients are analysed, as well as the bootstrap results of the indirect effect. When c and c’ coefficients are studied comparatively, it is observed that upon adding the mediator to the model, the c coefficient declines and its significance level changes. This indicates the partial mediation role of the variable of connection to nature. Additionally, due to the criticism directed at the causal step approach, the bootstrap results of the mediation role are calculated and reported as follows (see Table 3):

**Table 3:** Bootstrap results of indirect effect.

Scales	Effect	BootSE	BootLLCI	BootULCI
Connection to nature	−0.0231	0.0029	−0.0287	−0.0174

Based on the bootstrap results of mediation analysis, it can be seen that the confidence intervals do not include zero, meaning that the indirect effect is significant. This is considered to be more solid evidence of a mediation effect (Steffener, 2021; Zhang and Wang, 2008).

## Discussion

The results indicate that connection to nature partially mediates the relationship between behavioral problems and emotion regulation in preschool children. This mediating effect can be explained by several psychological mechanisms, including stress reduction (lowered cortisol levels), attentional restoration, and enhanced empathy, which together help children manage their emotional responses more effectively (Berman et al., 2008; de la Osa et al., 2023; Ernst and Stelley, 2024; Kuo, 2015; Kaplan and Kaplan, 1989; Pirchio et al., 2021; Sobko et al., 2018; Tyson Whitten et al., 2018). For instance, de la Osa et al. (2023) found that greater access to green spaces, particularly around schools, reduced anxiety in 539 children aged 3–11, while Arola et al. (2023) concluded that connection to nature decreases negative emotional states, improves mood and happiness, fosters self-efficacy, and promotes prosocial behaviors. These empirical findings provide a foundation for examining the specific mechanisms through which nature connection may mediate the effects of behavioral difficulties on emotional development.

Building upon previous work and addressing methodological limitations highlighted by Johnstone et al. (2022), the present partial mediation model demonstrates that connection to nature is not only linked to positive developmental outcomes but also offers a psychological pathway through which behavioral problems may influence emotion regulation. This underscores the potential of nature connection as a protective factor for children at risk and highlights the importance of considering nature-based interventions to support the development of emotion regulation skills.

Although nature connection plays a mediating role in the model examined in the study, the direct impact of behavioral problems on emotion regulation has not been completely eliminated. This suggests that other mechanisms also influence emotion regulation skills. The literature shows that these skills are affected by various factors, including individual factors (such as temperament traits and neurophysiological characteristics) and environmental factors (such as parent–child relationships and the parent’s way of regulating their own emotions) (Granic and Lamey, 2002; Hudson and Rao Pulla, 2023; İşcanoğlu et al., 2024). For example, children who experience behavioral challenges may find it more difficult to establish a healthy and meaningful connection with nature. Children with high levels of behavioral problems often struggle to maintain internal regulation and engage in harmonious interactions with their environment. Children exhibiting aggressive behaviors may have greater difficulty demonstrating flexibility in their actions due to reduced neural activity associated with emotion regulation (Lewis et al., 2006). Nevertheless, connecting with nature may provide a regulatory effect for children experiencing difficulties in emotion regulation by increasing cognitive flexibility and lowering cortisol levels (Berman et al., 2008). Beyond individual differences, environmental factors also shape children’s connection to nature and their emotion regulation skills.

Environmental factors, such as screen exposure, can influence children’s connection to nature. Excessive time spent in front of screens may trigger behavioral problems and reduce opportunities for children to benefit from the calming and balancing effects of nature (Qu et al., 2023; Xiang et al., 2022). Interventions that limit screen time while promoting nature-based activities can support the development of emotion regulation skills. In addition, understanding the impact of behavioral problems on emotion regulation underscores the importance of parent–child relationships. Rigid parenting interactions can hinder children’s ability to recognize, regulate, and appropriately express emotions (Granıc et al., 2012). Families of children with behavioral issues may adopt stricter, less flexible parenting styles, which can lead to conflicts and reduce outdoor activities with parents. Toshtemirova (2024) notes that spending time in nature enhances parent–child interactions, and activities such as hiking and gardening can strengthen these relationships. Overall, nature provides an environment that facilitates flexible, reciprocal, and supportive interactions, contributing to the development of children’s emotion regulation skills.

## Conclusion, limitations, and suggestions for future research

This study has determined that being connected with nature has an impact on the relationship between children’s behavioral problems and emotion regulation skills. It has been observed that connection to nature can serve not only as a tool for emotion regulation but also a promising protective factor for children with behavioral issues. These findings underscore the potential benefits of integrating nature-based activities into strategies that support children’s emotional development and may assist those with behavioral challenges.

There are some limitations in the study: First, the cross-sectional design of the study prevents us from making definitive causal inferences about the relationships between variables. Although the proposed mediation model is theoretically grounded and statistically sound, longitudinal or experimental designs are needed to establish causality. Second, the reliance solely on teacher reports for all measures, while ensuring a single-rater perspective, may introduce potential for reporter bias. Future studies could benefit from multi-informant approaches (e.g., adding parent reports or observational measures). Third, the model did not include several potential confounding variables that could influence the results, such as children’s temperament traits, family socioeconomic status in more detail, or parenting styles. The inclusion of these variables in future research could provide a more comprehensive understanding of the mechanisms at play. Furthermore, the sample consists of a specific age group; and the research was conducted in a limited geographical area. To increase the generalizability of the findings, samples should include different age groups and cover broader geographical regions. The relationships between behavioral problems, emotion regulation, and connection to nature should be examined across different age groups and through longitudinal studies to assess the persistence of these relationships.

The results obtained from the research indicate that children’s connection to nature can be strengthened through strategies such as integrating nature-based practices into school curricula, utilizing the forest school approach as a learning method, and increasing children’s access to green spaces. Detailed studies can be conducted to identify the factors that hinder children with behavioral issues from connecting with nature. Long-term studies can be carried out to examine the changes in children’s emotional regulation skills through intervention programs that enhance their connection to nature. The impact of children’s individual characteristics (such as gender, screen exposure time) on the relationships between behavioral issues, connection to nature, and emotional regulation skills can be explored in greater detail. The contribution of activities that children engage in with adults in their environment, such as spending time in nature with family and teachers conducting nature-based activities, to their emotional regulation skills can also be investigated.

## Data Availability

The original contributions presented in the study are included in the article/supplementary material, further inquiries can be directed to the corresponding author.
