# Correction: The mediating role of the connection with nature in the relationship between behavioral problems and emotion regulation in early childhood

**DOI:** 10.3389/fpsyg.2025.1741547

**Published:** 2025-12-08

**Authors:** Melike Aykut, H. Gözde Ertürk Kara

**Affiliations:** Kocaeli University, Izmit, Türkiye

**Keywords:** early childhood, connection to nature, emotion regulation, behavior problems, mediation analysis

Author Neslihan Tugçe Özyeter was erroneously included as an author in the published article. The correct author list reads:

Melike Aykut and H. Gözde Ertürk Kara^*^

Kocaeli University, Izmit, Türkiye

The author contributions statement was erroneously given as MA: Writing – original draft. HK: Writing – original draft, Writing – review & editing. NÖ: Data curation, Formal analysis, Methodology, Software, Validation, Writing – review & editing.

The correct author contributions statement is MA: Writing – original draft, Data curation, Formal analysis, Software; HK: Writing – original draft, Writing – review & editing, Methodology, Validation.

An incorrect **Funding** statement was provided: “The authors declare that financial support was received for the research and/or publication of this article. This article is derived from the first author's master's thesis at Kocaeli University.” The correct funding statement reads: “The authors declare that no financial support was received for the research and/or publication of this article. This article is derived from the first author's master's thesis at Kocaeli University under the supervision of the second author.”

The copyright for this article was incorrectly written as “© 2025 Aykut, Kara and Özyeter. This is an open-access article distributed under the terms of the **Creative Commons Attribution License (CC BY)**. The use, distribution or reproduction in other forums is permitted, provided the original author(s) and the copyright owner(s) are credited and that the original publication in this journal is cited, in accordance with accepted academic practice. No use, distribution or reproduction is permitted which does not comply with these terms.”

The correct statement is: “© 2025 Aykut and Ertürk Kara. This is an open-access article distributed under the terms of the **Creative Commons Attribution License (CC BY)**. The use, distribution or reproduction in other forums is permitted, provided the original author(s) and the copyright owner(s) are credited and that the original publication in this journal is cited, in accordance with accepted academic practice. No use, distribution or reproduction is permitted which does not comply with these terms.”

The original version of this article has been updated.

